# Supplementary material for: The role of somatosensory innervation of adipose tissues
Source: Nature. 2022 Aug 31;609(7927):569–74. doi: 10.1038/s41586-022-05137-7 (PMC9477745; doi:10.1038/s41586-022-05137-7)
Supplement: Supplementary file 1 — Full scans of the western blots for Extended Data Figure 7f. HSL was blotted after stripping of p-HSL. The red boxes indicate the areas that were cropped and displayed. [file 41586_2022_5137_MOESM1_ESM.pdf]

nature portfolio

https://doi.org/10.1038/s41586-022-05137-7

Supplementary information

The role of somatosensory innervation of adipose tissues

In the format provided by the authors and unedited

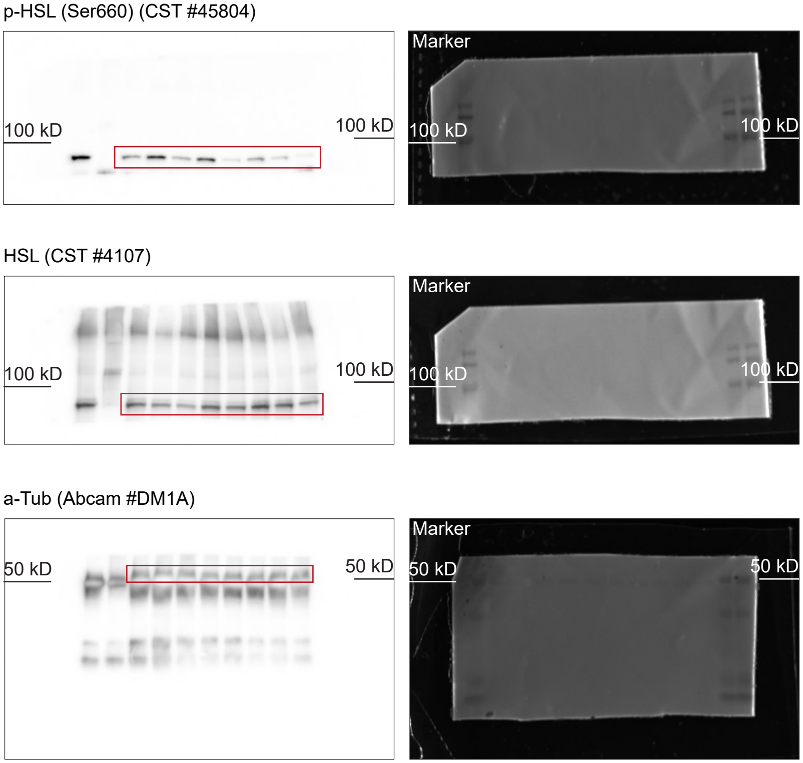

Supplementary Figure 1
Full scans of western blots for Extended Data Figure 7f. HSL is blotted after stripping of p-HSL. Red boxes indicate areas being cropped and displayed.
