# Supplementary material for: The role of somatosensory innervation of adipose tissues
Source: Nature. 2022 Aug 31;609(7927):569–74. doi: 10.1038/s41586-022-05137-7 (PMC9477745; doi:10.1038/s41586-022-05137-7)
Supplement: Supplementary file 2 — Reporting Summary [file 41586_2022_5137_MOESM2_ESM.pdf]

nature portfolio

Corresponding author(s): Ardem Patapoutian, Li Ye

Last updated by author(s): Jul 7, 2022

Reporting Summary

Nature Portfolio wishes to improve the reproducibility of the work that we publish. This form provides structure for consistency and transparency in reporting. For further information on Nature Portfolio policies, see our Editorial Policies and the Editorial Policy Checklist.

Statistics

For all statistical analyses, confirm that the following items are present in the figure legend, table legend, main text, or Methods section.

n/a	Confirmed	
☐	☒	The exact sample size (*n*) for each experimental group/condition, given as a discrete number and unit of measurement
☐	☒	A statement on whether measurements were taken from distinct samples or whether the same sample was measured repeatedly
☐	☒	The statistical test(s) used AND whether they are one- or two-sided<br>*Only common tests should be described solely by name; describe more complex techniques in the Methods section.*
☐	☒	A description of all covariates tested
☐	☒	A description of any assumptions or corrections, such as tests of normality and adjustment for multiple comparisons
☐	☒	A full description of the statistical parameters including central tendency (e.g. means) or other basic estimates (e.g. regression coefficient) AND variation (e.g. standard deviation) or associated estimates of uncertainty (e.g. confidence intervals)
☐	☒	For null hypothesis testing, the test statistic (e.g. *F*, *t*, *r*) with confidence intervals, effect sizes, degrees of freedom and *P* value noted<br>*Give P values as exact values whenever suitable.*
☒	☐	For Bayesian analysis, information on the choice of priors and Markov chain Monte Carlo settings
☐	☒	For hierarchical and complex designs, identification of the appropriate level for tests and full reporting of outcomes
☒	☐	Estimates of effect sizes (e.g. Cohen's *d*, Pearson's *r*), indicating how they were calculated

Our web collection on statistics for biologists contains articles on many of the points above.

Software and code

Policy information about availability of computer code

Data collection	FLUOVIEW 2.4.1.198 (OLYMPUS FV3000RS microscope), SmartSPIM GUI 2.1 (LifeCanvas Technnologies microscope), SmartSPIM Destriping and Stitching (2.0, LifeCanvas Technologies), SpinView (v2.5.0.80, FLIR BFS-U3 camera), BZ-X Viewer (Keyence BZ-X710 microscope), Bio-Rad CFX Manager (3.1, Biorad CFX384 qPCR machine)
Data analysis	samtools (v1.10), Salmon (v1.5.1), DESeq2 (v1.32.0), Metascape (v3.5), Fiji (v2.3.0; image projection and video making), IMARIS (v9.2.1; three-dimensional image rendering), Graphpad Prism (v9.3.1)<br>Custom codes used for sequencing analysis and FIJI analysis is deposited to https://github.com/yelabscripps.

For manuscripts utilizing custom algorithms or software that are central to the research but not yet described in published literature, software must be made available to editors and reviewers. We strongly encourage code deposition in a community repository (e.g. GitHub). See the Nature Portfolio guidelines for submitting code & software for further information.

Data

Policy information about availability of data

All manuscripts must include a data availability statement. This statement should provide the following information, where applicable:

- Accession codes, unique identifiers, or web links for publicly available datasets
- A description of any restrictions on data availability
- For clinical datasets or third party data, please ensure that the statement adheres to our policy

Bulk RNA-seq data is deposited under accession number GSE207664 (https://www.ncbi.nlm.nih.gov/geo/query/acc.cgi?acc=gse207664). The mice genome dataset (GRCm39 reference genome (Ensembl version 104) used for sequencing alignment can be accessed at http://uswest.ensembl.org/Mus_musculus/Info/Index.

All the numeric data in this study are included in the supplemental information. All other data supporting the findings of this study are too large for public deposit and are available from the corresponding author. Source data are provided with this paper.

Field-specific reporting

Please select the one below that is the best fit for your research. If you are not sure, read the appropriate sections before making your selection.

☒ Life sciences ☐ Behavioural & social sciences ☐ Ecological, evolutionary & environmental sciences

For a reference copy of the document with all sections, see nature.com/documents/nr-reporting-summary-flat.pdf

Life sciences study design

All studies must disclose on these points even when the disclosure is negative.

Sample size	No statistical methods were used to calculate the same size. The sample size was determined based on previous studies and literature in the field using similar experimental paradigms (Marshall KL et al. Nature, 2020; Lehnert BP et al. Cell, 2021; Bai L et al. Cell, 2019).
Data exclusions	No data were excluded, except mice with deteriorating health issues after surgery or during the experiment and mice with missed viral targeting assessed by histology.
Replication	All experiments were repeated at least twice with the same conclusions. All in vivo experiments were performed at least twice or grouped from two independent cohorts with the same conclusion, except long term high fat diet treatment was performed in one cohort for timing consideration. The exact number of replicated experiments are provided in the Statistics and reproducibility section.
Randomization	The individual animal's left or right sides were randomly assigned for unilateral treatment. Mice were randomly assigned for bilateral treatment.
Blinding	Data were collected blind, and post hoc registered to the treatment conditions and analyzed to prevent any bias.

Reporting for specific materials, systems and methods

We require information from authors about some types of materials, experimental systems and methods used in many studies. Here, indicate whether each material, system or method listed is relevant to your study. If you are not sure if a list item applies to your research, read the appropriate section before selecting a response.

Materials & experimental systems

n/a	Involved in the study	
☐	☒	Antibodies
☐	☒	Eukaryotic cell lines
☒	☐	Palaeontology and archaeology
☐	☒	Animals and other organisms
☒	☐	Human research participants
☒	☐	Clinical data
☒	☐	Dual use research of concern

Methods

n/a	Involved in the study	
☒	☐	ChIP-seq
☒	☐	Flow cytometry
☒	☐	MRI-based neuroimaging

Antibodies

Antibodies used	Anti-Perilipin-1 (Cell Signaling #9349, 1:400); Anti-TH-647 (BioLegend #818008, 1:300); Anti-beta-Tubulin (Abcam ab18207, 1:1000); Anti-Rabbit-488 (Jackson Immuno Research 711-546-152, 1;400); Anti-Rabbit-647 (Jackson Immuno Research 711-606-152, 1:400); Anti-p-HSL (S660) (Cell Signaling #45804, 1:1000), Anti-HSL (Cell Signaling #4107, 1:1000), Anti-α-Tubulin (Abcam #DM1A, 1:10000). Anti-Ucp1 (Abcam #ab10983, 1:200). Anti-rabbit HRP (Jackson Immuno Research 711-036-152, 1:10000), Anti-mouse HRP (Jackson Immuno Research 715-036-150, 1:10000), Anti-CGRP (Immunostar 24112, 1:1000).
Validation	Anti-Perilipin-1 (Cell Signaling #9349): mouse tissues, Maryanovich M., et al. Nat Med. (2018); Li C., et al. Nat Commun. (2020)<br>Anti-TH-647 (BioLegend, #818008): Non-conjugated form (clone 2/40/15, BioLegend #818001) validated on mouse tissues in: Ku T. et al, Nat Methods. (2020); Ku T. et al. Nat Biotechnol (2016).<br>Anti-beta-Tubulin (Abcam ab18207): mouse tissues, Walsh CM, et al. Elife (2019). Latremoliere A, et al. Cell Rep. (2018).<br>Anti-Ucp1 (Abcam #ab10983): mouse tissues, Chi J et al. Elife (2021).<br>Anti-p-HSL (S660) (Cell Signaling #45804): mouse tissues, Ding L et all, Nat Metab (2021).<br>Anti-HSL (Cell Signaling #4107): mouse tissues, Ding L et all, Nat Metab (2021).<br>Anti-α-Tubulin [DM1A] (Abcam #ab7291): HEK cell lines, Khan OM et al, Nat Commun (2021).<br>Anti-CGRP (Immunostar 24112): mouse tissues, Hill RZ et al. Nature. (2022).

Eukaryotic cell lines

Policy information about cell lines

Cell line source(s)	HEK293FT (Invitrogen R70007)
Authentication	HEK293FT was purchased from Invitrogen, no further authentication of line identity was performed.
Mycoplasma contamination	HEK293FT was purchased from Invitrogen, no further test for mycoplasma contamination was performed.
Commonly misidentified lines (See ICLAC register)	No commonly misidentified lines have been used.

Animals and other organisms

Policy information about studies involving animals; ARRIVE guidelines recommended for reporting animal research

Laboratory animals	Mice were group-housed in standard housing with 12:12h light:dark with ad libitum access to chow diet and water, with room temperature kept around 22C and humidity kept between 30-80% (not controlled), except for food intake measurement (single housed) and thermoneutral exposure experiments (30C). Mice of at least 6 weeks from the following strains were used for this study wild-type (WT) C57BL/6J (Jackson stock #000664), B6.Cg-Gt(ROSA)26Sortm9(CAG-tdTomato)Hze/J (Jackson stock #007909, Ai9), Pirt-cre (Kim, A. Y. et al. Cell 2008) , Scn10a-cre (Agarwal, N. et al. Genesis 2004). Both genders were used for anatomical mapping studies, while male mice were used for in vivo functional experiments.
Wild animals	The current study did not utilize wild animals.
Field-collected samples	The current study did not utilize field-collected samples.
Ethics oversight	All experimental protocols were approved by The Scripps Research Institute Institutional Animal Care and Use Committee (Animal protocol 18-0001, 08-0136) and were in accordance with the guidelines from the NIH.

Note that full information on the approval of the study protocol must also be provided in the manuscript.
